# Antibacterial effect of *Rubus chingii* flower extract against multidrug-resistant bacteria

**DOI:** 10.6026/97320630018938

**Published:** 2022-10-31

**Authors:** Amir Saeed

**Affiliations:** 1 Department of Clinical Laboratory Sciences, College of Applied Medical Sciences, University of Hail, P.O. Box 2440, UAE; 2 Department of Medical Microbiology, Faculty of Medical Laboratory Sciences, University of Medical Sciences & Technology, Khartoum, Sudan

**Keywords:** *Rubus chingii*, flower, extract, antibacterial, *K. pneumoniae*, *S. aureus*, *E. coli.*

## Abstract

*Rubus chingii* is a well reputed member of Chinese traditional medicine system and is used for managing different ailments since historic times. The present report elucidates the growth impeding effect of R. chingii flower extract against multidrug resistant Escherichia coli, Klebsiella pneumoniae and Staphylococcus aureus. The extracts were prepared using standard Soxhlet extraction method using ethanol, methanol and acetone as a solvent. The extracts were further subjected to agar streak method and the stains that showed their sensitivity were further evaluated for minimum inhibitor concentration (MIC) assessment through TCC method. Subsequently the MIC was further used for well diffusion assay. All the strains used in the study showed their sensitivity towards R. chingii flowers extract in respective solvents. Highest antibacterial activity was seen against *E. coli.* and *S. aureus* whereas the lowest activity was recorded against *K. pneumoniae*. Thus the study reported herewith provided an insight into the antibacterial efficacy of R. chingii flower extract against MDR *E. coli.*, *S. aureus* and *K. pneumoniae*.

## Background:

Rubus chingii Hu, commonly known as immature Chinese raspberry, served to be a source of immense dietary and nutritional food from ancient times. Previously several important classes of bioactive constituents namely terpenoids, organic acids and flavonoids have been identified from various parts of this plant [1]. These biologically active classes of phytocompounds are commonly associated with diverse pharmacological attributes of R. chingii which includes anti-inflammatory, anti-aging and antimicrobial activities [2]. Subsequently, reports in recent times have elucidated the anticancer efficacy of R. chingii against several cancers in vitro [3- 4]. Inspite of considerable advancement in organic chemistry during the past decade, approximately 25% of the pharmaceutical market in developed nations is full of medicines whose bioactive constituents are extracted naturally from plants [6]. Secondary metabolites extracted from plants are a group of biologically functional chemical compounds possessing several pharmacological attributes which in turn is a boon for mankind. Therefore, identification and investigation of these bioactive phytoconstituents can positively impact the global pharmaceutical market by balancing the limited abilities of synthetic bioactive constituents. Several antibiotics serve a limited effectual life and the development of resistance among individuals due to their over prescription has been a global concern [7]. This situation is further worsened by the mindset of individuals from developing nations who intend to have autonomy on their medical issues resulting in being self-medication as a common happening [8]. Genus Rubus, falls under the family Rosaceae, which is important fruit widely distributed through the entire Northern Hemisphere [9]. More than 700 different plant species are reported to fall under this family of which some prominent ones are R. chingii, R. parvifolius and R. rosifolius among others [10]. R. chingii is an important plant whose fruits are commonly referred in Chinese as "Fu-Pen-Zi". Due to its intrinsic pharmacological and nutritional attributes R. chingii has been a common plant in Chinese traditional system of medicines [11]. Therefore, it is of interest to describe the antibacterial attributes of different solvent extracts of R. chingii flowers against MDR E. coli, S. aureus and K. pneumoniae was investigated.

## Materials and Methods:

### Sample collection:

Fresh flowers from R. chingii plant were collected from the garden. The flowers were rinsed firstly with normal tap water and then with deionized to remove the dirt. The flowers were then subjected to shade drying for at least a week and in this period were constantly monitored for any sort of contamination. After drying, the plant material was powdered using mortar pestle and sieved through 8" sieve. The resultant plant material was stored at 4°C in polystyrene containers.

### Extract preparation:

The powered flower extract of R. chingii was weighed (20 g) and thereafter extracted in ethanol, methanol and acetone through Soxhlet's extraction for nearly 72 hours. The resultant extract was thereafter subjected to vacuum evaporation to obtain dried plant extract. The extract was stored at -20°C till further use in amber colored eppendorf.

### Test cultures:

The multidrug resistant cultures used in the study, namely E. coli, S. aureus and K. pneumoniae, were obtained from ATCC. Nutrient agar was used in slants for storing the stated cultures in slants at 4°C.

### Preparation of inoculums:

All the bacterial strain used in the present report was cautiously sub-cultured for 24 h on nutrient agar slants. 106 CFU/ml corresponding to 25% of UV-Vis light transmittance at 560 nm in NaCl (0.85% w/v) were used for inoculation. The turbidity of suspension was adjusted using McFarland standard which was constituted by BaCl2 (50 µl; 1.7% BaCl2.2H2O w/v) and 0.18 M H2SO4 (9.95 ml) under constant stirring. The McFarland standard was protected from being evaporated by concealing in an air tight container or test-tube [12].

### Antimicrobial Assay:

The bacterial growth impeding potential of R. chingii flower extracts were initially scrutinized using agar streak. These strains were observed to be inhibited in their growth by R. chingii flower extracts and were therefore evaluated for their minimum inhibitory concentration (MIC). The extracts with their observed MICs were subsequently evaluated for their antimicrobial efficacy using well diffusion assay.

### Preliminary antibacterial evaluation:

The extracts were initially evaluated for their sensitivity against all the MDR strains, as said above, through the agar streak method. 1 mg/ml of each extract was mixed with 15 ml of sterile agar butts and the resulting mixture was poured into the sterilized petri dish. After solidification of the mixture, bacterial suspension was inoculated on the solidified agar Petri dishes and was incubated for 24 h at 37°C. The results were interpolated in terms of positive and/or negative growth of the bacterial cultures as presented in Table 1(see PDF).

### Estimation of Minimum Inhibitory Concentration (MIC):

Micro-well diffusion methodology was employed for estimating the MIC of R. chingii flower extracts in nutrient broth. 2, 3, 5-triphenyltetrazolium chloride or TCC (0.01% w/v) served as an indicator for bacterial growth. 5 µl of bacterial inoculums along with 95 µl broth was supplemented in each well of a 96-well plate.

Subsequently, R. chingii flower extracts, serially diluted in respective solvents, were supplemented in each well followed by TCC. The 96-well plate was further subjected to incubation for 24 h at 37°C. Solvent served as the negative control whereas streptomycin and penicillin were the positive control. Bacterial growth is reported to convert TCC into deep red colored formazan which in turn is an indicator of bacterial cell viability [13]. The interpolations of the observations were made depending on the presence and/or absence of red formazan and are presented in Table 2(see PDF).

### Well diffusion method:

R. chingii flower extracts were further evaluated for its antibacterial competency through a well diffusion assay. Sub-culturing was performed a day prior to experimentation on agar slants and the preparation of inoculum was accomplished as stated above. Subsequently, the inoculum was added to 20 ml of sterile molten nutrient agar, the mixture was homogenized and poured into petri dishes. Wells were cut thereafter using sterile metallic well cutter once the agar in the plate was solidified. Wells were then filled with 100 µl of extract in respective solvents, and Penicillin along with Streptomycin was used as positive control. The MIC value of each extract in respective solvent was used during the assay as shown in Figure 1(see PDF). The plate was subjected to 24 h of incubation at 37°C. Vernier calipers were used to measure zone of inhibition as reported in Table 3(see PDF).

## Results:

Results of agar streak method are shown in Table 1(see PDF). The ethanolic extract of R. chingii flowers showed better antimicrobial efficacy for E. coli and K. pneumoniae, while methanolic extract showed better efficacy against E. coli and S. aureus. However, acetone extract showed better efficacy only against K. pneumoniae. The MIC of R. chingii flower extracts was further evaluated using TCC. It was observed that with an increase in the concentration of R. chingii flower ethanolic extract the viability of MDR bacterial cultures was considerably reduced due to reduction in the formation of red formazan. Thus, it is evident from the results that R. chingii flower extracts exerted a significant bactericidal effect against E. coli, S. aureus and K. pneumoniae. However, the efficiency of the extracts was variable against different MDR bacterial cultures tested as shown in Table 2(see PDF). Furthermore, assessment of zone of inhibition revealed two main observations which included the wells not showing any zone of inhibition and the wells with clear demarcation for zone of inhibition. These observations may be explicitly inferred to be arising due to absence and presence of any bactericidal efficacy of R. chingii flower extracts. The zone of inhibitions, observed at MICs of all the R. chingii flower extracts, was variable among the entire tested microorganism as shown in Table 3(see PDF).

## Discussion:

Since the beginning of human origin, plants have served to be an important source of biologically active chemical moieties that exerts chemotherapeutic action against several human ailments. Among all the advanced stages of drug development, the preliminary in vitro analysis serves to be the initial yet important criteria. The current study is focused towards investigating the bactericidal function of R. chingii flower extracts in different solvents namely ethanol, methanol and acetone against MDR bacteria. The observations from the present study initially elucidated that the all the extracts of R. chingii flowers exhibited substantial bactericidal efficiency against MDR E. coli, S. aureus and K. pneumoniae in terms of MIC values. Importantly zone of inhibition was also seen in cases of all the different solvents; however, the range of inhibition varied among all the tested MDR strains. Highest zone of inhibition was seen with ethanolic extract where 36.21 mm of inhibition was observed in E. coli. Lowest zone of inhibition i.e. 22.67 mm was seen in case of K. pneumoniae also subjected to ethanol extract of R. chingii flowers. The zone of inhibition was also compared with Streptomycin and Penicillin which served as positive control and the results elucidated the greater and/or comparable effects of different solvent extracts. It is now established that bioactivity of any plant or its parts is due to presence of phyto compounds and/or secondary metabolites. The solubility of these phyto compounds depends largely on the solvent which was also seen in the present study since the acetone extract of R. chingii flower showed comparably lesser bactericidal activity against all the three bacteria cultures used in this study. Another important factor contributing during the assessment of the zone of inhibition is the ability of the solvent used for extracting the bioactive phytoconstituents to penetrate and diffuse through the nutrient media used in the study [14-17]. The results of this study clearly indicated the bactericidal potential of R. chingii flower extracts in various solvents. These can further be fractionated for the identification of bactericidal constituents that can be further employed for further research and development of therapeutics against stated microbes. Furthermore, our results also elucidated that R. chingii flower extracts exhibit greater and/or comparable bactericidal potency to that of positive control.

## Conclusions:

The above results clearly indicated that R. chingii flowers intrinsically possess considerable anti-bacterial efficacy and hold the potential for further detailed investigation to understand and elucidate their mechanistic anti-bacterial action. Moreover, the fractionation-based study shall also be warranted before explicitly establishing R. chingii flower extracts as anti-bacterial therapeutics.
